# Correlation between treatment-related symptom burden and psychological flexibility in patients with cervical cancer and their combined correlational association with emotional distress

**DOI:** 10.3389/fpsyt.2026.1895713

**Published:** 2026-09-04

**Authors:** Jiao Ma, Yan Peng, Qin Chen, Xinying He, Haixiang Wang

**Affiliations:** 1 Department of Nursing, Women's Hospital of Nanjing Medical University, Nanjing Women and Children’s Healthcare Hospital, Nanjing, Jiangsu, China; 2 Nursing Department, Hai' an Clinical College, Medical College of Yangzhou University, Nantong, China; 3 Department of Critical Care Medicine, The Affiliated Wuxi People’s Hospital of Nanjing Medical University, Wuxi People’s Hospital, Wuxi Medical Center, Nanjing Medical University, Wuxi, Jiangsu, China

**Keywords:** cervical cancer, correlation, emotional distress, psychological flexibility, symptom burden

## Abstract

**Objective:**

To explore the correlation between treatment-related symptom burden and psychological flexibility in patients with cervical cancer, as well as the combined correlational association of these two factors with patients’ emotional distress, so as to provide a theoretical basis for formulating targeted psychological intervention strategies in clinical practice.

**Methods:**

A cross-sectional survey was conducted. A total of 216 patients with cervical cancer who received treatment in our hospital from June 2025 to January 2026 were selected as the research subjects. Data were collected using a general information questionnaire, the Memorial Symptom Assessment Scale (MSAS), the Multidimensional Psychological Flexibility Inventory-24 (MPFI-24), the Acceptance and Action Questionnaire-II (AAQ-II), and the Hospital Anxiety and Depression Scale (HADS). Pearson correlation analysis was used to explore the relationships among symptom burden, psychological flexibility, and different dimensions of emotional distress. Hierarchical regression analysis was performed to examine the correlational association of the interaction between symptom burden and psychological flexibility on emotional distress.

**Results:**

Showed that the total HADS score of the 216 patients with cervical cancer was (14.32 ± 4.56) points, with an emotional distress detection rate of 48.15%. Pearson correlation analysis indicated that the total symptom burden score and its subscale scores were positively correlated with the anxiety and depression subscale scores of HADS (*P* < 0.05). The total MPFI-24 score, its subscale scores, and the AAQ-II score were negatively correlated with the anxiety and depression subscale scores of HADS (*P* < *0.*05). In addition, the total symptom burden score was negatively correlated with the total MPFI-24 score and the AAQ-II score (*P* < 0.05). Hierarchical regression analysis further showed that, after controlling for general demographic and disease-related variables, symptom burden and psychological flexibility were independent correlates of emotional distress, and their interaction had a significant correlational association on emotional distress (*P* < 0.05).

**Conclusion:**

Patients with cervical cancer experience a significant treatment-related symptom burden and relatively low levels of psychological flexibility, and both factors jointly affect patients’ emotional states. In clinical practice, comprehensive intervention measures aimed at reducing symptom burden and improving psychological flexibility may help alleviate emotional distress and enhance the quality of life of these patients.

## 1 Introduction

Cervical cancer is a common malignant tumor of the female reproductive system worldwide, ranking fourth in the incidence of female malignant tumors. The incidence in China is increasing year by year, and it shows a trend toward younger age groups (1). At present, comprehensive diagnosis and treatment modes such as surgery, radiotherapy, chemotherapy, and targeted therapy have significantly improved the 5-year survival rate of patients with cervical cancer. However, a series of physiological manifestations, such as pain, fatigue, gastrointestinal reactions, and urogenital symptoms, occur during the treatment process and cause patients to bear a heavy symptom burden (2). This symptom burden not only directly impairs patients’ physical function, but also easily induces anxiety, depression, and other emotional problems. Emotional distress, in turn, aggravates the perception of symptoms, forming a vicious cycle of “symptom burden-emotional distress,” which seriously reduces treatment compliance and quality of life (3) in patients. Therefore, clarifying the correlation mechanism between symptom burden and emotional distress in patients with cervical cancer, and identifying key targets for intervention, are of great significance for improving patient prognosis.

Psychological flexibility is the core concept of acceptance and commitment therapy (ACT), and refers to the ability of individuals to flexibly adjust cognitive, emotional, and behavioral responses according to their own values and goals when facing difficulties. It is mainly reflected in acceptance of negative experiences, cognitive dissociation, self-background, clear value and commitment to action (4). The Acceptance and Action Questionnaire (AAQ) is a classic tool for assessing psychological flexibility. The simplified version, AAQ-II, can effectively reflect the degree of avoidance of psychological discomfort. The short version of the Multidimensional Psychological Flexibility Inventory (MPFI-24) can comprehensively assess the level of psychological flexibility from multiple dimensions (5). It has been confirmed that psychological flexibility plays an important role in symptom management and emotion regulation in patients with cancer. The level of psychological flexibility in patients with breast cancer and lung cancer is negatively correlated with anxiety and depression symptoms, and can buffer the adverse effects of disease-related stress on emotion (6). However, there is a lack of research on the association between symptom burden and psychological flexibility in patients with cervical cancer, and the joint correlational pattern linking the two indicators to emotional distress remains unclear.

Most studies focus on breast and lung cancer. Cervical cancer patients have unique urogenital adverse reactions and disease stigma, and relevant joint association evidence remains insufficient.

This cross-sectional study explored correlations among symptom burden, psychological flexibility and emotional distress, and tested the moderating effect of psychological flexibility. Three directional hypotheses were put forward: Hypothesis 1: Symptom burden positively correlates with emotional distress; psychological flexibility negatively correlates with emotional distress. Hypothesis 2: Symptom burden and psychological flexibility are independent correlates of emotional distress after controlling covariates. Hypothesis 3: Psychological flexibility weakens the correlation between symptom burden and emotional distress.

## 2 Research methods

### 2.1 Study subjects

From June 2025 to January 2026, convenience sampling was used to select patients with cervical cancer treated in our hospital as the research subjects. Sample size estimation was based on the principles of hierarchical regression analysis. Considering the number of independent variables (general information, 10 items; symptom burden, 6 items; psychological flexibility, 8 items) and a 10% sample attrition rate, the sample size was determined to be no less than 200 cases, and 216 cases were ultimately included in this study.

**Figure 1:**
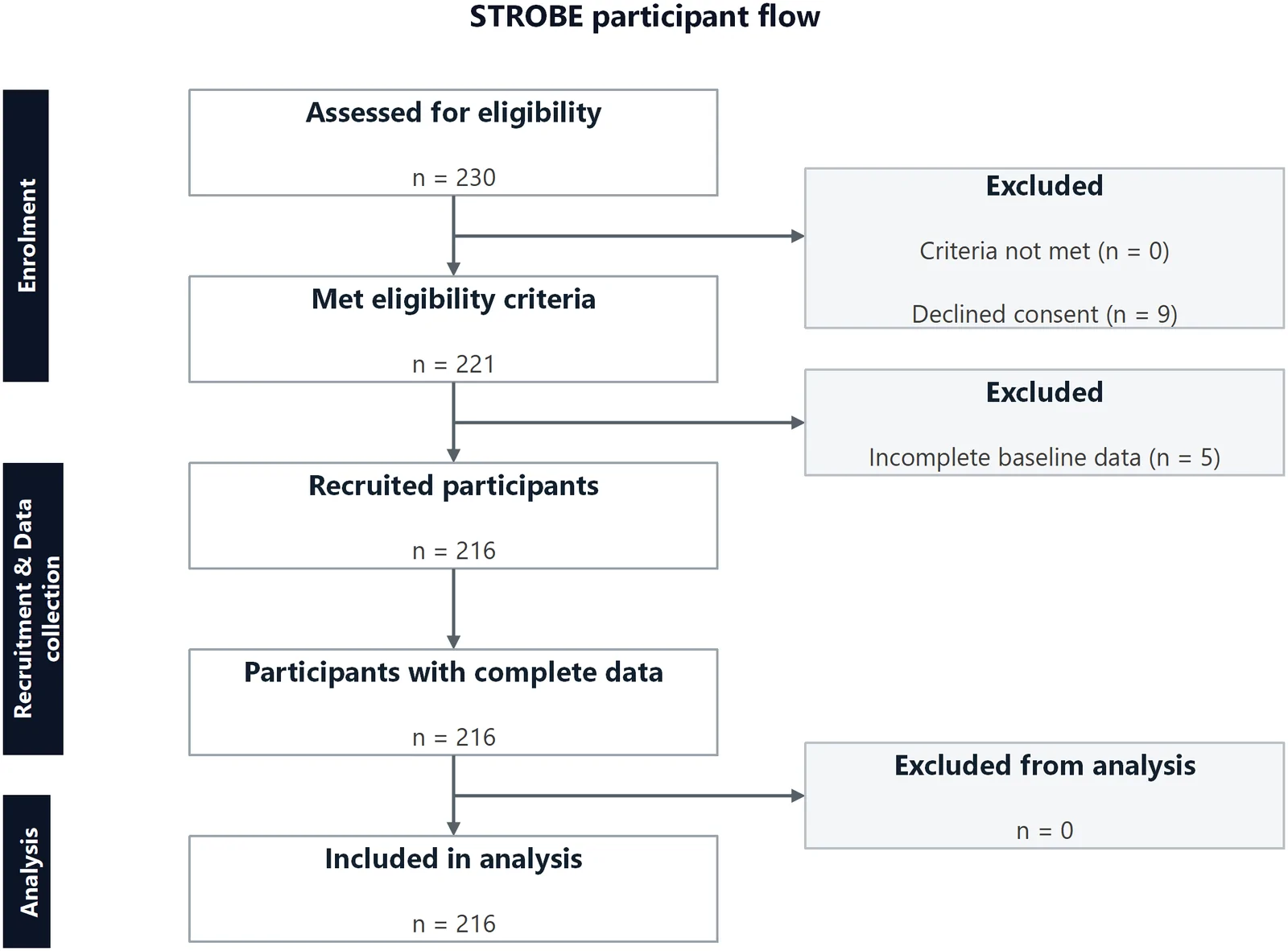
STROBE-compliant participant flow chart of this cross-sectional study.

A total of 230 patients met initial screening criteria and were invited to participate. Among them, 9 patients declined participation due to fatigue and poor physical tolerance, and 5 were excluded for incomplete treatment records, leaving a final valid sample of 216(see **Figure 1**). A standard STROBE participant flow diagram was added to visually display the screening process.

Inclusion criteria: (1) cervical cancer confirmed by histopathological examination; (2) receiving anti-tumor therapy such as surgery, radiotherapy, chemotherapy, or targeted therapy; (3) age ≥18 years, clear consciousness, and ability to understand and cooperate in completing the questionnaire; (4) voluntary participation in this study with signed informed consent. Exclusion criteria: (1) presence of other malignant tumors or serious organic diseases such as heart, liver, or kidney disease; (2) cognitive dysfunction, mental illness, or communication disorders; (3) inability to complete the survey due to critical illness; (4) previous history of psychotherapy. The study was reviewed and approved by the Medical Ethics Committee of Nanjing Women and Children’s Healthcare Hospital (Decision Number: PJ-2025KY017-001). Informed consent was obtained from all participants. All procedures strictly complied with the Declaration of Helsinki to protect participants’ rights, privacy, and welfare.

### 2.2 Research tools

#### 2.2.1 General information questionnaire

The questionnaire was self-designed by the investigators according to the purpose of the study. It included demographic and sociological data (age, education level, marital status, occupation, and family monthly income) and disease-related data (clinical stage, pathological type, treatment plan, and treatment duration).

#### 2.2.2 Memorial symptom assessment scale

The MSAS is an internationally widely used cancer symptom assessment tool, which aims to comprehensively quantify the frequency, severity, and interference (7) of disease- and treatment-related symptoms in cancer patients. The Chinese version of the MSAS has been translated and tested for reliability and validity, and is suitable for assessing the burden (8) of treatment-related symptoms in cancer patients in China. The scale includes two dimensions: physical symptoms (24 items) and psychological symptoms (6 items), with a total of 30 items. Each item is evaluated from three aspects: presence of symptoms (0 = no, 1 = yes), severity of symptoms (1 = mild, 2 = moderate, 3 = severe), and degree of symptom interference (1 = mild, 2 = moderate, 3 = severe). The total symptom burden score is calculated as (total severity score + total interference score)/number of items, with higher scores indicating a heavier symptom burden. Reliability and validity testing of the Chinese version in lung cancer patients showed that the Cronbach’s α coefficient of the total scale was 0.90, with values of 0.88 for the physical symptom dimension and 0.76 for the psychological symptom dimension. The content validity index (CVI) ranged from 0.86 to 1.00.

#### 2.2.3 Multidimensional psychological flexibility inventory-24

The MPFI-24 comprehensively assesses psychological flexibility across multiple dimensions, which compensates for the shortcomings (9) of single-dimension scales. The Chinese version of the MPFI-24 has been revised and verified for reliability and validity, and is suitable for measuring psychological flexibility in the Chinese population (10). The scale includes six dimensions: acceptance, cognitive dissociation, focus on the present, self-as-context, clarity of values, and commitment to action, with four items in each dimension, for a total of 24 items. A 7-point Likert scoring method is used, ranging from 1 = not at all consistent to 7 = completely consistent. Higher total scores indicate higher levels of psychological flexibility. The Chinese version of the scale has been validated in adolescents and adults. Cronbach’s α coefficient of the total scale was 0.91, and coefficients for each dimension ranged from 0.76 to 0.87. Confirmatory factor analysis supported the six-factor structure (CFI = 0.92, TLI = 0.91, RMSEA = 0.05).

#### 2.2.4 Hospital anxiety and depression scale

The HADS was used to screen patients for anxiety and depression. It includes two dimensions, anxiety and depression, each containing seven items (11). A 4-point scoring method (0–3 points) is applied, with each dimension scoring from 0 to 21 points. A score ≥8 points indicates the presence of significant anxiety or depression. In cancer patients, the Cronbach’s α coefficients of the Chinese version were 0.80 for the anxiety dimension and 0.78 for the depression dimension, with test-retest reliability ranging from 0.73 to 0.76. The validity indices met psychometric requirements (12).

#### 2.2.5 Acceptance and action questionnaire-II

The AAQ-II is a revised version of the original AAQ, with improved psychometric properties (13). The Chinese version of the AAQ-II has been sinicized and is suitable for the assessment of psychological rigidity in the Chinese population (14). The scale consists of seven items and adopts a 7-point scoring method, ranging from 1 = completely inconsistent to 7 = completely consistent. Higher total scores indicate greater psychological rigidity, that is, lower levels of psychological flexibility. The Chinese version of the scale has been validated in college students and clinical samples. Cronbach’s α coefficient was 0.84, test-retest reliability was 0.72, and confirmatory factor analysis supported a single-factor structure (CFI = 0.95, RMSEA = 0.06).

### 2.3 Research methods

#### 2.3.1 Data collection

Trained researchers explained the purpose, content, and precautions of the study in detail to patients who met the inclusion criteria. After informed consent was obtained, the questionnaires were distributed. For patients with a low education level or those unable to complete the questionnaire independently, researchers asked the questions verbally according to unified instructions and recorded the patients’ responses truthfully. All participants were divided into two subgroups: self-completed questionnaire group and researcher-assisted interview group. Data collection mode was recorded as a covariate to control potential bias caused by different survey forms in subsequent regression analysis. A total of 230 questionnaires were distributed, and 216 valid questionnaires were returned, yielding an effective response rate of 93.91%.

#### 2.3.2 Statistical analysis

SPSS 29.0 software was used for data analysis. Measurement data were expressed as mean ± standard deviation, and count data were expressed as frequency (percentage). Pearson correlation analysis was conducted to examine the relationships between symptom burden, psychological flexibility, and each dimension of emotional distress. Harman single-factor test was performed to examine common method bias (CMB) from self-reported questionnaires; the maximum single-factor variance explained was less than 40%, indicating no serious common method variance in this study. Demographic and clinical variables were selected as control variables based on existing domestic and foreign oncology psychology literature, which have been confirmed to correlate with cancer patients’ emotional distress. The criterion for independent variables entering the regression equation was *P* < *0.*05, and the exclusion criterion was *P* > 0.10. Data collection mode was added into Step 1 of hierarchical regression as an additional control variable to eliminate its confounding effect on emotional distress scores. A value of *P* < 0.05 was c*o*nsidered statistically significant.

## 3 Results

### 3.1 General information

The average age of the 216 patients with cervical cancer was (52.36 ± 10.24) years (range, 28–75 years). The education level was mainly junior high school or below (58.33%). Most patients were married (82.41%). Farmers and unemployed individuals accounted for the majority (65.74%). A total of 52.78% had a monthly household income of less than 3000 yuan. The clinical stage was mainly II–III (73.15%). The pathological type was predominantly squamous cell carcinoma (80.56%). The main treatment modality was chemoradiotherapy (56.48%). The treatment duration ranged from 1 to 12 months, with an average of (5.32 ± 2.16) months. Based on data collection modes, all participants were divided into self-completed group (n=132) and researcher-assisted interview group (n=84). Supplementary subgroup comparison showed small but statistically significant differences in MSAS, HADS and MPFI-24 total scores between the two subgroups (all *P* < 0.05), confirming that survey mode affected self-reported scores, which was adjusted as a covariate in hierarchical regression.

### 3.2 Scores of symptom burden, psychological flexibility, and emotional distress in patients with cervical cancer

The total MSAS symptom burden score of the 216 patients with cervical cancer was (2.18 ± 0.65), including (2.25 ± 0.68) for the physical symptom dimension and (2.03 ± 0.72) for the psychological symptom dimension. The total MPFI-24 score was (86.32 ± 15.45). The total AAQ-II score was (28.45 ± 7.32). The total HADS score was (14.32 ± 4.56), with an anxiety factor score of (7.28 ± 2.31) and a depression factor score of (7.04 ± 2.45). Regarding emotional distress, 92 cases (42.59%) had anxiety, 88 cases (40.74%) had depression, and 104 cases (48.15%) had emotional distress.

### 3.3 Correlation analysis of symptom burden, psychological flexibility, and emotional distress in patients with cervical cancer

Pearson correlation analysis showed that the total symptom burden score and the physical and psychological symptom dimension scores of the MSAS were positively correlated with the anxiety and depression factor scores of the HADS (*r* = 0.326–0.589, all *P* < 0.01). The total score and each dimension score of the MPFI-24 were negatively correlated with the anxiety and depression factor scores of the HADS (*r* = –0.412 to –0.635, all *P* < 0.01). The total AAQ-II score was positively correlated with the anxiety and depression factor scores of the HADS (*r* = 0.435–0.578, all *P* < 0.01). In addition, the total MSAS symptom burden score was negatively correlated with the total MPFI-24 score (*r* = –0.387, *P* < 0.01) and positively correlated with the total AAQ-II score (*r* = 0.452, *P* < 0.01). The specific correlation coefficients are shown in **Table 1**.

**Table 1:** Correlation analysis of symptom burden, psychological flexibility, and emotional distress in patients with cervical cancer.

Variables	1 Total MSAS score	2. Physical symptoms	3. Psychological symptoms	4. MPFI-24 total score	5. Total AAQ-II score	6. HADS anxiety score	7. HADS depression score
1. Total MSAS score	1	–	–	–	–	–	–
2. Physical symptoms	0.892^**^	1	–	–	–	–	–
3. Psychological symptoms	0.765^**^	0.543^**^	1	–	–	–	–
4. MPFI-24 total score	-0.387^**^	-0.354^**^	-0.421^**^	1	–	–	–
5. Total AAQ-II score	0.452^**^	0.418^**^	0.503^**^	-0.689^**^	1	–	–
6. HADS anxiety score	0.589^**^	0.526^**^	0.635^**^	-0.578^**^	0.578 **	1	–
7. HADS depression score	0.542^**^	0.498^**^	0.601^**^	0.635 * *	0.435 * *	0.792 * *	1

**P < *0.*01; MSAS, Memorial Symptom Assessment Scale; MPFI-24, Multidimensional Psychological Flexibility Questionnaire; short version; AAQ-II, Acceptance and Action Questionnaire; second edition; HADS, Hospital Anxiety and Depression Scale.

### 3.4 Hierarchical regression analysis of symptom burden and psychological flexibility on emotional distress

HADS scores were used as the dependent variable, and hierarchical regression analysis was performed. In the first step, general demographic and disease-related variables and data collection mode were included as control variables. In the second step, the total symptom burden score (MSAS) and psychological flexibility indicators (MPFI-24 total score and AAQ-II total score) were included as independent variables. In the third step, the interaction term between the total symptom burden score and the total MPFI-24 score, and the interaction term between the total symptom burden score and the total AAQ-II score, were included as independent variables (all interaction terms were mean-centered to avoid multicollinearity).

The regression results showed that the control variables in the first step explained 12.3% of the variance in emotional distress (*F* = 3.258, *P* = 0.005). Among these variables, family monthly income (*β* = –0.213, *P* = 0.008) and clinical stage (*β* = 0.186, *P* = 0.015) were significant correlates of emotional distress. When symptom burden and psychological flexibility were included in the second step, the explanatory power of the model increased significantly to 48.6% (*ΔR^2^* = 0.363, *ΔF* = 45.287, *P* < *0.*001). The total symptom burden score (*β* = 0.352, *P* < 0*.001*), the total MPFI-24 score (*β* = –0.421, *P* < 0.001), an*d* the total AAQ-II score (*β* = 0.325, *P* < 0.001) were all independent correlates of emotional distress. After inclusion of the interaction terms in the third step, the explanatory power of the model further increased to 52.1% (*ΔR^2^* = 0.035, *ΔF* = 9.864, *P* = 0.002). The in*terac*tion between symptom burden and MPFI-24 total score had a significant correlational association on emotional distress (*β* = –0.186, *P* < 0.01), and the interaction between symptom burden and AAQ-II total score was also significant (*β* = 0.154, *P* < 0.05). The detailed results are shown in **Table 2**, with 95% confidence intervals supplemented for all *β* coefficients and interaction terms.

**Table 2:** Hierarchical regression analysis of symptom burden and psychological flexibility on emotional distress.

Model	Independent variable	Beta	*SE*	*t*	*P*	*R^2^*	*Δ R squared*	*Δ F*	*95%CI*
Step 1	Constant term	10.258	2.134	4.807	<0.001	0.123	0.123	3.258	[6.065, 14.451]
	Age	-0.087	0.052	-1.673	0.097				[-0.189, 0.015]
	Level of education	-0.121	0.078	-1.551	0.123				[-0.274, 0.032]
	Monthly household income	-0.213	0.082	-2.597	0.008				[-0.374, -0.052]
	Clinical staging	0.186	0.075	2.480	0.015				[0.038, 0.334]
	Treatment options	0.095	0.068	1.397	0.164				[-0.039, 0.229]
	Data collection mode	0.104	0.064	1.625	0.105				[-0.022, 0.230]
Step 2	MSAS total score	0.352	0.065	5.415	<0.001	0.486	0.363	45.287	[0.224, 0.480]
	MPFI-24 total score	-0.421	0.061	-6.902	<0.001				[-0.541, -0.301]
	AAQ-II total score	0.325	0.072	4.514	<0.001				[0.183, 0.467]
Step 3	MSAS×MPFI-24	-0.186	0.063	-2.952	0.004	0.521	0.035	9.864	[-0.309, -0.063]
	MSAS×AAQ-II	0.154	0.071	2.169	0.032				[0.014, 0.294]

In Step 1, general demographic and disease-related variables (age, education level, marital status, family monthly income, clinical stage, and treatment plan) were included. Only key variables are presented in the table. MSAS, Memorial Symptom Assessment Scale; MPFI-24, Multidimensional Psychological Flexibility Questionnaire, short version; AAQ-II, Acceptance and Action Questionnaire, second edition.

To further clarify the interaction effects, patients were divided into a high psychological flexibility group (MPFI-24 ≥ 86 points, n = 108) and a low psychological flexibility group (MPFI-24 < 86 points, n = 108) based on the median MPFI-24 total score (86 points). Simple slope analyses were then conducted to examine the relationship between symptom burden and emotional distress in each group. The results showed that in the low psychological flexibility group, symptom burden had a stronger correlational association with emotional distress (*β* = 0.528, *t* = 8.352, *P* < *0.*001). In contrast, in the high psychological flexibility group, the correlational slope of symptom burden on emotional distress was significantly reduced (*β* = 0.176, *t* = 2.*684, P* = 0.008), indicating that higher psychological flexibility weakened the positive correlational link between symptom burden and emotional distress.

Supplemental CMB test result: Harman single-factor test showed the maximum explained variance of the first unrotated factor was 28.7%, lower than the critical threshold of 40%, suggesting no severe common method bias in self-reported data.

## 4 Discussion

Emotional health problems in patients with cervical cancer during treatment require urgent attention. This study found that emotional distress was relatively common in patients with cervical cancer, with high detection rates of anxiety, depression, and related emotional problems. This finding is closely related (15) to the disease characteristics of the study population. A large proportion of the patients included in this study were in the middle or advanced stages of disease and were receiving combined radiotherapy and chemotherapy. These patients not only face psychological pressure related to disease progression, but also endure various physical discomforts associated with radiotherapy and chemotherapy.

From the perspective of symptom burden, patients with cervical cancer generally showed an upper-middle level, with physiological symptoms having a particularly prominent impact. This is highly consistent with the clinical characteristics of cervical cancer treatment. While controlling disease progression, conventional treatment methods such as surgery, radiotherapy, and chemotherapy inevitably damage normal tissues and lead to a series of specific and nonspecific physiological symptoms. Persistent physical discomfort not only directly reduces patients’ quality of life, but also further aggravates psychological stress through amplification of symptom perception. As an important psychological resource for coping with adversity, psychological flexibility was generally low in patients with cervical cancer. Continuous negative experiences related to disease and treatment may cause patients to develop rigid patterns of thinking and behavior, making it difficult to flexibly adjust coping strategies according to actual circumstances and thereby impairing their ability (16) to adapt to the disease.

The strong association between symptom burden and emotional distress further reflects the complex mechanism underlying their interaction. Persistent somatic symptoms continuously consume psychological resources, increase feelings of helplessness and hopelessness, and induce or exacerbate anxiety, depression, and other emotional problems. Conversely, negative emotions can heighten sensitivity to physical discomfort through neuroendocrine regulation, forming a vicious (17) cycle of “enhanced symptom perception-emotional deterioration.” This interaction has also been observed in other cancer populations, suggesting that combined interventions targeting symptom management and emotional regulation have broad relevance across cancer types. Notably, all correlation coefficients obtained in this study were moderate (*r* = 0.326–0.589) despite statistical significance, which indicated that symptom burden and psychological flexibility only explained partial variation in emotional distress; multiple unmeasured influencing factors may exist in the real clinical environment.

At the same time, the negative association between psychological flexibility and emotional distress supports the core concept of acceptance and commitment therapy (ACT). Individuals with higher psychological flexibility tend to accept disease-related negative experiences with a more open attitude, reduce the constraints of negative thinking through cognitive dissociation, and focus on valued life goals and treatment adherence, thereby buffering emotional stress. Notably, a significant association was also observed between symptom burden and psychological flexibility. Patients experiencing heavier symptom burden over long periods were more likely to exhibit lower psychological flexibility. This finding suggests that clinical interventions should address both physical symptom relief and the enhancement (18) of psychological resources.

The interaction mechanism revealed by the hierarchical regression analysis provides a clear target for clinical psychological intervention. After controlling for demographic, clinical characteristics and data collection mode, both symptom burden and psychological flexibility were independent correlates of emotional distress, and their interaction indicated that psychological flexibility significantly moderated the effect of symptom burden on emotional distress. Specifically, in patients with a low level of psychological flexibility, the negative correlational link between symptom burden and emotional state was stronger. These patients often adopt rigid coping styles, such as avoidance and repression, which are ineffective in alleviating negative emotions. In contrast, patients with higher psychological flexibility are able to adjust their cognition and behavior more flexibly, view symptoms as stage-specific responses during treatment, actively seek social support, and engage in positive coping strategies such as relaxation training, thereby weakening the correlational link between symptom burden and emotional distress (19). This finding is consistent with related studies in patients with lung cancer, suggesting that enhancing psychological flexibility may serve as an effective entry point for emotional interventions across different cancer types. Based on these findings, a dual-track intervention model of “somatic symptom intervention + psychological flexibility training” should be developed in clinical practice. Medical staff can integrate core techniques of acceptance and commitment therapy to help patients enhance present-moment awareness through mindfulness training, guide them to clarify core treatment and life goals through values clarification, and encourage the adoption of positive and healthy behaviors through commitment-based action training. At the same time, targeted symptom management measures, such as pain control and interventions for gastrointestinal reactions, can be implemented to achieve synergistic improvement in both physical and psychological domains. For example, psychological support groups for patients with cervical cancer can be organized regularly, with systematic psychological flexibility training led by professional psychotherapists. In addition, oncology nurses can be invited to provide education on symptom management knowledge and skills, thereby helping patients relieve physical discomfort and enhance psychological adaptability (20). The hierarchical regression results showed that each step significantly increased the explained variance, yet the interaction term only brought a small incremental *R²* of 0.035. Although this increment was numerically small, the interaction effect remained statistically significant. According to the viewpoint of Shivappa et al (21), small but stable moderating effects can produce cumulative beneficial effects in large-sample long-term clinical psychological intervention programs, which deserves clinical attention. However, the dual-track intervention strategy proposed in this paper is only a theoretical inference based on cross-sectional correlational data. At present, there is insufficient empirical evidence to verify its efficacy among cervical cancer patients. Relevant intervention recommendations can only be put forward as research hypotheses, and subsequent randomized controlled trials are needed for verification.

This study has several limitations that should be addressed in future research. First, the cross-sectional design adopted in this study could only identify correlational links among variables and could not clarify causal relationships or temporal sequences of symptom burden, psychological flexibility and emotional distress, and bidirectional interactions and reverse causality between these indicators could not be ruled out; longitudinal repeated measurement tracking studies are required to sort out dynamic changes of variables throughout the whole treatment course. In terms of sampling, single-center convenience sampling limited the participants to patients receiving treatment in one tertiary hospital, which inevitably introduced selection bias, and the research conclusions could not be generalized to patients treated in primary medical institutions, patients from different regions or groups with different cultural backgrounds; multi-center stratified sampling design is recommended for follow-up studies. In terms of measurement tools, all study indicators were collected via self-reported questionnaires, which may lead to recall bias and social desirability bias. Although we carried out Harman single-factor test to control common method variance statistically, subjective reporting deviation could not be completely eliminated, and subsequent research can adopt multi-source evaluation by combining objective physiological indicators such as cortisol level and clinician scoring scales. In addition, two different questionnaire administration forms including independent self-completion and researcher-assisted interview were used in this survey. Although we took data collection mode as a covariate into regression analysis to adjust its confounding effect, subgroup comparisons still confirmed that different survey modes would affect participants’ self-reported scores, and unified standardized self-report tools should be adopted in future investigations. Finally, limited by questionnaire length and on-site investigation time, this study failed to measure several important psychosocial factors including social support, coping strategies and psychological resilience, which may act as confounding variables or mediating variables in the observed correlational pathways. Subsequent researchers can incorporate these indicators into the research framework and construct more comprehensive influencing factor models via structural equation modeling.

## 5 Conclusion

Patients with cervical cancer experience a heavy burden of treatment-related symptoms and low levels of psychological flexibility, and these factors jointly correlate with their emotional state. In clinical practice, comprehensive intervention strategies combining symptom relief and psychological flexibility training can only be regarded as theoretical hypotheses derived from cross-sectional data, and further interventional trials are needed to verify their efficacy for alleviating emotional distress and improving patients’ quality of life.

## Data Availability

The raw data supporting the conclusions of this article will be made available by the authors, without undue reservation.
